# Association of triglyceride-glucose index with clinical outcomes in patients with acute ischemic stroke receiving intravenous thrombolysis

**DOI:** 10.1038/s41598-022-05467-6

**Published:** 2022-01-31

**Authors:** Emma M. S. Toh, Amanda Y. L. Lim, Chua Ming, Leonard L. L. Yeo, Ching-Hui Sia, Bryce W. Q. Tan, Aloysius S. T. Leow, Jamie S. Y. Ho, Bernard P. L. Chan, Vijay Kumar Sharma, Benjamin Y. Q. Tan

**Affiliations:** 1 Yong Loo Lin School of Medicine, National University of Singapore, 10 Medical Drive, Singapore, 117597 Singapore; 2 Division of Endocrinology, Department of Medicine, National University Health System, 1E Kent Ridge Road, Singapore, 119228 Singapore; 3 Division of Neurology, Department of Medicine, National University Health System, 1E Kent Ridge Road, Singapore, 119228 Singapore; 4 Department of Cardiology, National University Heart Centre Singapore, 1E Kent Ridge Road, Singapore, 119228 Singapore; 5 Department of Medicine, National University Health System, 1E Kent Ridge Road, Singapore, 119228 Singapore; 6 School of Clinical Medicine, Addenbrooke’s Hospital, University of Cambridge, Hills Rd, Cambridge, CB2 0SP UK

**Keywords:** Stroke, Endocrine system and metabolic diseases, Cerebrovascular disorders

## Abstract

Intravenous tissue plasminogen activator (tPA) remains the cornerstone of recanalization therapy for acute ischemic stroke (AIS), albeit with varying degrees of response. The triglyceride-glucose (TyG) index is a novel marker of insulin resistance, but association with outcomes among AIS patients who have received tPA has not been well elucidated. We studied 698 patients with AIS who received tPA from 2006 to 2018 in a comprehensive stroke centre. TyG index was calculated using the formula: ln[fasting triglycerides (mg/dL) × fasting glucose (mg/dL)/2]. TyG index was significantly lower in patients that survived at 90-days than those who died (8.61 [Interquartile Range: 8.27–8.99] vs 8.76 [interquartile range: 8.39–9.40], p = 0.007). In multivariate analysis, TyG index was significantly associated with 90-day mortality (OR: 2.12, 95% CI: 1.39–3.23, p = 0.001), poor functional outcome (OR: 1.41 95% CI: 1.05–1.90, p = 0.022), and negatively associated with early neurological improvement (ENI) (OR: 0.68, 95% CI: 0.52–0.89, p = 0.004). There was no association between TyG index and symptomatic intracranial hemorrhage. ‘High TyG’ (defined by TyG index ≥ 9.15) was associated with mortality, poor functional outcomes and no ENI. In conclusion, the TyG index, a measure of insulin resistance, was significantly associated with poorer clinical outcomes in AIS patients who received tPA.

## Introduction

While intravenous tissue plasminogen activator (IV tPA) remains the mainstay of acute ischemic stroke (AIS) treatment and its use continues to rise^1^, overall functional outcomes of AIS patients have not improved even with more successful recanalization^2^, where up to three-quarters of patients experience unfavorable outcomes^3^. Furthermore, patients also risk having symptomatic intracranial hemorrhage (SICH) following IV tPA^4^. These highlight a need for greater risk stratification to guide clinical decision making in thrombolysis. Meanwhile, it is estimated that a quarter of the world population suffers from metabolic syndrome^5^, but despite such a high burden of disease, there are a lack of specific guidelines to risk-stratify post-thrombolysis AIS patients with metabolic syndrome^6^. Furthermore, conflicting evidence of the relationship between metabolic syndrome (and its components) and outcomes post-thrombolysis further add to the lack of clarity^7–9^. Thus, a reliable and practical measure of metabolic disease should be utilized to re-assess this relationship with post-thrombolysis stroke outcomes.

While Insulin Resistance (IR), part of the underlying pathophysiology of disorders related to metabolic syndrome^10^, has shown correlation with poor post-stroke outcomes^11^, current measures of IR, such as the hyperglycemic clamp, the hyperinsulinemic-euglycemic clamp test (HEC) and homeostatic model assessment of insulin resistance (HOMA-IR), are not practical in a clinical setting. Alternative measures of IR that reflect various components of metabolic syndrome have been proposed, but among these, the Triglyceride-Glucose (TyG) Index was shown to be superior to other similar markers for identifying IR risk and risk of developing diabetes^12,13^. The TyG Index has recently been established as a reliable, cost-effective and easily accessible surrogate marker for IR^14,15^, and performed better than HOMA-IR in estimating IR when compared to the hyperglycemic clamp^16^. Furthermore, the TyG index performed superiorly to fasting glucose and triglycerides alone in predicting development of Type 2 diabetes^17^, and notably increased the risk of cardio-cerebrovascular disease^18^, suggesting the TyG index may potentially be a good marker of metabolic disease to predict post-stroke outcomes.

While the TyG index has demonstrated predictive effects in major adverse cardiovascular event outcomes in patients with acute coronary disease undergoing percutaneous coronary intervention^19^, and was superior to fasting glucose and hemoglobin A1c (HbA1C) in this setting^20^, it has not been well investigated in stroke populations. While previous authors have found an association between incidence of AIS and TyG index^21,22^, limited studies have reported the effect of the TyG index on post-stroke clinical outcomes or recurrence^23–25^, and the relationship of the TyG index in a thrombolyzed patient cohort has been studied scarcely. The only two studies that have investigated the effect of IR on AIS patients’ response to thrombolysis both utilized HOMA-IR and were limited by sample size or analyzed only non-diabetic patients^26,27^. Hence, further studies in a current, larger cohort of patients are required to establish the significance of a relationship between the TyG index and post-thrombolysis stroke outcomes. Therefore, we sought to understand the association of the TyG index, as a reflection of IR, in AIS patients who underwent thrombolysis.

## Methods

### Study design

This is a retrospective cohort study of consecutive patients who received IV tPA from September 2006 to June 2018 and was approved by the local institutional review board. Patients were assessed by a neurologist to be eligible to receive intravenous thrombolysis according to institutional protocol and American Heart Association/American Stroke Association guidelines at a standard dose of 0.9 mg/kg body weight^6^. All stroke patients who were thrombolyzed underwent standard non-contrast head Computed Tomography and Computed Tomography brain and neck angiography. Patients with valid laboratory readings of their fasting triglycerides and venous glucose levels, taken within 24 h of their AIS admission were included. Patients who were deemed unsuitable for IV tPA were excluded from the study. Other baseline demographics, clinical parameters and ischemic stroke characteristics were collected and tabulated within 24 h of AIS admission. Diabetes Mellitus (DM) was defined as pre-existing diagnosis of diabetes mellitus or an admitting fasting blood glucose level greater than or equal to 7.0 mmol/L (126 mg/dl) or an HbA1c greater than or equal to 6.5%. The presence of Large Vessel Occlusion (LVO) was defined as occlusions of the first and second segment of the middle cerebral artery (MCA), the Internal Carotid Artery (ICA) and as well as its terminus, tandem occlusions involving ICA-MCA, or occlusion of the basilar artery. The severity of stroke at presentation was assessed using the National Institute of Health Stroke Scale (NIHSS)^28^, recorded as admitting NIHSS. This assessment was made by credentialed nurses as part of the acute stroke response team. The Trial of Org 10172 in Acute Stroke Treatment (TOAST) criteria was used by the treating stroke neurologist to classify stroke subtypes^29^. A cut-off for TyG index of 9.15 was calculated using the internationally recognised triglyceride cut-off for hypertriglyceridemia of 150 mg/dL (1.7 mmol/L)^30^ and the cut-off of fasting glucose of 126 mg/dL (7.0 mmol/L), which was selected based on the fasting glucose cut-off for the diagnosis of diabetes^31^. All procedures were performed in accordance with the relevant guidelines.

The TyG Index was calculated using the formula: ln[fasting triglycerides (mg/dL) × fasting glucose (mg/dL)/2]^32^. The primary outcome studied was all-cause 90-day mortality. The secondary outcomes included poor functional outcome, for which the dichotomous definition of poor functional outcome was defined as having a 90-day modified Rankin Scale (mRS) of 3–6. The 90-day mRS was evaluated during the follow-up visit to the stroke clinic, and if patients were not able to attend the follow-up visit physically, mRS was evaluated via telephone call instead. Other secondary outcomes included early neurological improvement (ENI), defined as a decrease in NIHSS score by 4 points or more within 24 h or complete resolution of neurological deficits at 24 h, and SICH, based on the European Cooperative Acute Stroke Study (ECASS) II definition: any type of intracerebral hemorrhage on any post-treatment imaging after the start of thrombolysis and increase of ≥ 4 NIHSS points from baseline, or from the lowest value within 7 days, or leading to death^33^.

### Data analysis

Statistical tests were performed using R version 4.0.2 (R Core Team, Vienna, Austria), and a p-value of < 0.05 was considered statistically significant. We performed statistical analysis using Mann–Whitney *U* test for non-normally distributed continuous variables, and Chi-square analysis for categorical variables. All continuous variables were non-normally distributed as determined by visual histogram and Shapiro–Wilk statistical tests, and median and interquartile range (IQR) were reported for each variable. A logistic regression model was then performed to identify independent predictors of mortality, 90-day functional outcome, ENI and SICH. Covariates that were selected a priori for variable adjustment were age, hypertension, NIHSS recorded at presentation (admitting NIHSS), and LVO status. The adjusted multivariate logistic regression model was then employed to evaluate the associations of TyG index with the respective outcomes and presented as adjusted odds ratio (OR) with 95% confidence intervals (CI).

Characteristics of patients with ‘Low TyG’ and ‘High TyG’ indices were then compared using ≥ 9.15 as a cut-off value, including the distributions of 90-day functional outcome (measured by mRS) between the two groups of patients. Receiver-operating characteristic (ROC) curve analysis was performed in R to determine the performance of the TyG index and the cut-off value in predicting the primary outcome of mortality. Logistic regression was again performed to determine the relationships between ‘High TyG’ and ‘Low TyG’ and dichotomous outcomes including mortality, ENI and SICH. For functional outcome, ordinal shift analysis was carried out to compare associations between patient cohorts with ‘Low TyG’ and ‘High TyG’ indices and ordinal functional outcome (mRS 0–6), where multivariate ordinal logistic regression was presented as OR with 95% CI. After analyzing the main cohort, subgroup analysis was conducted to investigate the relationship of TyG index in patients with and without diabetes respectively. Similarly, baseline characteristics and multivariate logistic regression were carried out for both cohorts of patients with and without diabetes.

Multivariate Imputation by Chained Equations (MICE) in R was employed for missing baseline data to conduct multivariate regression analyses. Variables with originally more than 25% missing data were excluded from multivariate analysis but were used as predictors in imputation. Missing baseline data was imputed with MICE. The multiple imputation method used was Predictive Mean Matching (PMM) for scale variables and logistic regression for categorical variables. Predictors used in the imputation included age, race, gender, smoking status, hypertension, hyperlipidemia, DM, atrial fibrillation (AF), fasting glucose, random plasma glucose, HbA1c, serum total cholesterol, low-density lipoprotein cholesterol (LDL), high-density lipoprotein cholesterol (HDL), triglycerides, the cholesterol:HDL (cholHDL) ratio, serum aspartate transaminase (AST), alanine transaminase (ALT), admitting hematocrit, total white blood cells, lymphocytes, neutrophils, platelets, admitting NIHSS, NIHSS at 2 h and 24 h, systolic blood pressure on admission, 2 h and 24 h, diastolic blood pressure on admission, 2 h and 24 h, baseline mRS, mRS at discharge, 90-day mRS, TOAST classification, SICH, onset-to-treatment time for tPA administration, and LVO status. 30 imputed datasets were created and estimates from the multivariate analysis were pooled.

### Ethics approval and consent to participate

Ethics approval was obtained from National Healthcare Group—Domain Specific Review Board (NHG DSRB Reference: 2010/00509) where approval for informed consent was waived as this was patient level data.

## Results

### Baseline characteristics

A total of 698 patients satisfying the eligibility criteria were included in the final analysis. The baseline characteristics of the study population is summarised in Table 1. The patient cohort had a median age of 65 years (IQR: 55–76), was predominantly male (n = 420, 61.5%) and of Chinese ethnicity (n = 424, 68.7%), and the median admitting NIHSS was 14 (IQR: 8–21). With respect to stroke subtypes, there were large-vessel atherosclerotic strokes (n = 157, 30.4%), cardioembolic strokes (n = 198, 38.3%), small-vessel occlusion (n = 71, 13.7%), strokes of other determined etiology (n = 10, 1.9%) and stroke of unknown etiology (n = 81, 15.7%). A large proportion of patients had hypertension (n = 428, 80.8%) and hyperlipidemia (n = 326, 70.7%), and a smaller proportion of patients had DM (n = 214, 30.7%), were smokers (n = 108, 34.6%) and had AF (n = 124, 38.9%). The median TyG index was 8.62 (IQR: 8.29–9.01). There were n = 63 deaths (9.1%) and n = 308 patients (44.6%) experienced poor functional outcome (mRS 3–6) ninety days after stroke onset. ENI was experienced by n = 389 patients (59.7%) and n = 32 patients (4.6%) suffered a SICH. Median TyG index was significantly lower in survivors at 90-days than those who died (8.61 [IQR: 8.27–8.99] vs 8.76 [IQR: 8.39–9.40], p = 0.007) (Table 1).

**Table 1:** Characteristics of all patients that underwent IV-tPA, and comparison between patients stratified by mortality.

Variable	Total (*N* = 698)	Mortality		*p*
		Alive *n* = 627	Dead *n* = 63	
Age (years, median [IQR])	65.00 [55.00, 76.00]	64.00 [54.75, 75.00]	78.00 [65.00, 86.0]	< 0.001
Male (n/total, %)	420/683 (61.5)	386/612 (63.1)	30/63 (47.6)	0.023
**Ethnicity (n/total, %)**				0.172
Chinese	424/617 (68.7)	375/550 (68.2)	43/59 (72.9)	
Malay	118/617 (19.1)	103/550 (18.7)	14/59 (23.7)	
Indian	34/617 (5.5)	32/550 (5.8)	1/59 (1.7)	
Others	41/617 (6.6)	40/550 (7.3)	1/59 (1.7)	
**Comorbidities (n/total, %)**				
Smoking	108/312 (34.6)	106/292 (36.3)	2/16 (12.5)	0.094
Hypertension	428/530 (80.8)	382/480 (79.6)	42/44 (95.5)	0.018
Hyperlipidemia	326/461 (70.7)	287/414 (69.3)	34/41 (82.9)	0.1
Atrial fibrillation	124/319 (38.9)	109/288 (37.8)	11/24 (45.8)	0.579
Diabetes mellitus	214/698 (30.7)	179/627 (28.5)	32/63 (50.8)	< 0.001
**Glucose level**				
Fasting glucose (mmol/L, median [IQR])	5.90 [5.20, 7.30]	5.90 [5.20, 7.10]	7.40 [6.05, 9.25]	< 0.001
HbA1c (%, median [IQR])	5.90 [5.60, 6.30]	5.85 [5.50, 6.20]	5.95 [5.60, 6.50]	0.241
**Lipid parameters**				
Total cholesterol (mmol/L, median [IQR])	4.68 [3.92, 5.36]	4.74 [4.02, 5.42]	4.19 [3.56, 5.01]	< 0.001
LDL (mmol/L, median [IQR])	2.93 [2.25, 3.51]	2.98 [2.30, 3.56]	2.43 [1.98, 3.01]	< 0.001
HDL (mmol/L, median [IQR])	1.13 [0.97, 1.34]	1.14 [0.97, 1.34]	1.12 [0.98, 1.33]	0.875
Triglycerides (mmol/L, median [IQR])	1.13 [0.84, 1.58]	1.12 [0.84, 1.58]	1.22 [0.80, 1.64]	0.526
Cholesterol HDL ratio (median [IQR])	4.05 [3.27, 4.96]	4.12 [3.31, 5.04]	3.66 [3.08, 4.31]	0.003
TyG index (median [IQR])	8.62 [8.29, 9.01]	8.61 [8.27, 8.99]	8.76 [8.39, 9.40]	0.007
**Stroke parameters**				
Admitting NIHSS (median [IQR])	14.0 [8.0, 21.0]	13.0 [7.0, 20.0]	22.0 [19.0, 26.0]	< 0.001
NIHSS at 24 h (median [IQR])	6.0 [2.0, 15.0]	5.0 [2.0, 13.0]	24.0 [16.5, 27.0]	< 0.001
Onset-to-treatment time (min, median [IQR])	155.50 [118.00, 204.50]	157.00 [118.75, 207.00]	154.50 [109.25, 185.75]	0.126
Admitting systolic BP (mmHg, median [IQR])	152.00 [135.75, 168.00]	152.00 [135.00, 168.00]	153.00 [136.00, 170.00]	0.848
Admitting diastolic BP (mmHg, median [IQR])	83.00 [73.00, 92.00]	82.00 [73.75, 92.00]	83.00 [69.00, 92.00]	0.549
Large vessel occlusion (n/total, %)	420/643 (65.3)	360/576 (62.5)	54/60 (90.0)	< 0.001
**TOAST (n/total, %)**				0.124
Large-artery atherosclerosis	157/517 (30.4)	144/469 (30.7)	11/41 (26.8)	
Cardioembolism	198/517 (38.3)	172/469 (36.7)	21/41 (51.2)	
Small-vessel occlusion (lacunae)	71/517 (13.7)	69/469 (14.7)	2/41 (4.9)	
Stroke of other determined etiology	10/517 (1.9)	8/469 (1.7)	2/41 (4.9)	
Stroke of unknown etiology	81/517 (15.7)	76/469 (16.2)	5/41 (12.2)	
Mortality (n/total, %)	63/690 (9.1)	0/627 (0.0)	63/63 (100.0)	< 0.001
**90-day mRS (n/total, %)**				< 0.001
0	159/690 (23.0)	159/627 (25.4)	0 (0.0)	
1	148/690 (21.4)	148/627 (23.6)	0 (0.0)	
2	75/690 (10.9)	75/627 (12.0)	0 (0.0)	
3	77/690 (11.2)	77/627 (12.3)	0 (0.0)	
4	136/690 (19.7)	136/627 (21.7)	0 (0.0)	
5	32/690 (4.6)	32/627 (5.1)	0 (0.0)	
6	63/690 (9.1)	0 (0.0)	63 (100.0)	
Early neurological improvement (n/total, %)	389/652 (59.7)	371/596 (62.2)	16/51 (31.4)	< 0.001
Symptomatic intracranial hemorrhage (n/total, %)	32/697 (4.6)	21/626 (3.4)	10/63 (15.9)	< 0.001

*n* number, *total* number of non-missing values of each variable, *p* p-value of Pearson’s Chi-squared test for categorical variables and Mann–Whitney U test for continuous variables, *IQR* interquartile range, *mmol/L* millimoles per liter, *min* minutes, *mmHg *millimeters of mercury, *LDL* low density lipoprotein, *HDL* high density lipoprotein, *TyG index* triglyceride-glucose index, *NIHSS* National Institutes of Health Stroke Scale, *OTT* onset-to-treatment time, *BP* blood pressure, *TOAST* trial of org 10172 in acute stroke treatment, *mRS* modified Rankin scale.

The median TyG index was significantly lower in those who experienced ENI (8.56 [IQR: 8.26–8.92] vs 8.69 [IQR: 8.31–9.07], p = 0.007), but there was no significant difference in median TyG index observed between those with good and poor functional outcomes (8.62 [IQR: 8.29–8.98] vs 8.62 [IQR: 8.28–9.08], p = 0.623). There was no significant difference in median TyG index between those who experienced SICH compared to those who had not (8.59 [IQR: 8.22–8.97] vs 8.62 [IQR: 8.29–9.01], p = 0.823) (Table 2).

**Table 2:** Comparison between patients stratified by poor functional outcome, early neurological improvement and symptomatic intracranial hemorrhage.

Variable	Poor functional outcome			Early neurological improvement			Symptomatic intracranial hemorrhage		
	mRS (0–2) *n* = 382	mRS (3–6) *n* = 308	*p*	ENI *n* = 389	No ENI *n* = 263	*p*	No SICH *n* = 665	SICH *n* = 32	*p*
Age (median in years [IQR])	61.00 [52.00, 70.00]	74.00 [60.00, 81.00]	< 0.001	64.00 [54.00, 76.00]	66.00 [56.00, 77.00]	0.094	65.00 [55.00, 76.00]	69.00 [58.50, 76.00]	0.386
Male (n/total, %)	259/375 (69.1)	157/300 (52.3)	< 0.001	237/379 (62.5)	157/259 (60.6)	0.685	402/650 (61.8)	17/32 (53.1)	0.422
**Ethnicity (n/total, %)**			0.005			0.286			0.330
Chinese	218/340 (64.1)	200/269 (74.3)		224/340 (65.9)	168/235 (71.5)		400/587 (68.1)	24/29 (82.8)	
Malay	67/340 (19.7)	50/269 (18.6)		74/340 (21.8)	36/235 (15.3)		114/587 (19.4)	4/29 (13.8)	
Indian	24/340 (7.1)	9/269 (3.3)		20/340 (5.9)	14/235 (6.0)		34/587 (5.8)	0 (0.0)	
Others	31/340 (9.1)	10/269 (3.7)		22/340 (6.5)	17/235 (7.2)		39/587 (6.6)	1/29 (3.4)	
**Comorbidities (n/total, %)**									
Smoking	76/178 (42.7)	32/130 (24.6)	0.002	69/181 (38.1)	34/106 (32.1)	0.366	106/287 (36.9)	2/24 (8.3)	0.009
Hypertension	224/286 (78.3)	200/238 (84.0)	0.122	237/296 (80.1)	165/197 (83.8)	0.360	408/501 (81.4)	20/28 (71.4)	0.287
Hyperlipidemia	183/250 (73.2)	138/205 (67.3)	0.205	186/258 (72.1)	124/170 (72.9)	0.935	308/432 (71.3)	18/28 (64.3)	0.564
Atrial fibrillation	52/164 (31.7)	68/148 (45.9)	0.014	73/184 (39.7)	40/107 (37.4)	0.793	116/293 (39.6)	8/25 (32.0)	0.594
Diabetes mellitus	91/382 (23.8)	120/308 (39.0)	< 0.001	94/389 (24.2)	103/263 (39.2)	< 0.001	203/665 (30.5)	11/32 (34.4)	0.791
**Glucose level**									
Fasting glucose (mmol/L, median [IQR])	5.70 [5.00, 6.40]	6.50 [5.50, 8.20]	< 0.001	5.70 [5.10, 6.60]	6.30 [5.50, 8.05]	< 0.001	5.90 [5.20, 7.30]	6.40 [5.57, 7.55]	0.118
HbA1c (%, median [IQR])	5.80 [5.50, 6.20]	5.90 [5.60, 6.40]	0.003	5.80 [5.50, 6.10]	5.90 [5.60, 6.40]	0.011	5.90 [5.60, 6.30]	5.95 [5.70, 6.20]	0.588
**Lipid parameters**									
Total cholesterol (mmol/L, median [IQR])	4.82 [4.21, 5.47]	4.47 [3.70, 5.28]	< 0.001	4.70 [4.00, 5.36]	4.72 [3.92, 5.44]	0.691	4.70 [3.94, 5.36]	4.28 [3.79, 5.44]	0.447
LDL (mmol/L, median [IQR])	3.10 [2.43, 3.61]	2.70 [2.03, 3.37]	< 0.001	2.97 [2.30, 3.51]	2.97 [2.28, 3.59]	0.774	2.93 [2.26, 3.51]	2.70 [2.02, 3.53]	0.434
HDL (mmol/L, median [IQR])	1.13 [0.96, 1.33]	1.13 [0.98, 1.35]	0.228	1.13 [0.96, 1.35]	1.14 [0.98, 1.32]	0.740	1.13 [0.96, 1.34]	1.18 [1.06, 1.34]	0.242
Triglycerides (mmol/L, median [IQR])	1.21 [0.87, 1.64]	1.07 [0.78, 1.46]	0.001	1.13 [0.86, 1.54]	1.11 [0.80, 1.59]	0.814	1.13 [0.84, 1.58]	1.12 [0.77, 1.44]	0.304
Cholesterol HDL ratio (median [IQR])	4.26 [3.42, 5.19]	3.75 [3.10, 4.72]	< 0.001	4.06 [3.30, 4.98]	4.13 [3.28, 5.02]	0.923	4.06 [3.29, 4.98]	3.72 [2.99, 4.50]	0.079
TyG index (median [IQR])	8.62 [8.29, 8.98]	8.62 [8.28, 9.08]	0.623	8.56 [8.26, 8.92]	8.69 [8.31, 9.07]	0.007	8.62 [8.29, 9.01]	8.59 [8.22, 8.97]	0.823
**Stroke onset parameters**									
Admitting NIHSS (median [IQR])	10.0 [6.0, 16.0]	19.0 [13.0, 23.0]	< 0.001	15.0 [9.0, 21.0]	11.0 [6.0, 20.0]	< 0.001	14.0 [8.0, 20.0]	21.0 [16.0, 22.0]	< 0.001
NIHSS at 24 h (median [IQR])	3.0 [1.0, 6.0]	15.0 [9.0, 21.0]	< 0.001	4.0 [1.0, 9.0]	14.0 [6.0, 21.0]	< 0.001	6.0 [2.0, 14.0]	20.0 [12.5, 23.0]	< 0.001
Onset-to-treatment time (min, median [IQR])	160.00 [120.50, 208.50]	153.00 [114.00, 200.00]	0.146	151.00 [113.00, 201.00]	161.00 [123.75, 208.00]	0.074	156.00 [117.00, 205.50]	149.00 [133.00, 188.00]	0.940
Admitting systolic BP (mmHg, median [IQR])	151.00 [135.00, 165.00]	154.00 [136.75, 172.25]	0.065	150.00 [135.00, 165.00]	155.00 [135.25, 170.75]	0.084	152.00 [135.50, 167.50]	161.50 [146.00, 171.00]	0.265
Admitting diastolic BP (mmHg, median [IQR])	82.00 [74.00, 90.00]	83.0 [72.00, 93.00]	0.643	81.00 [71.50, 90.00]	85.00 [75.00, 93.00]	0.013	83.00 [73.00, 92.00]	83.50 [71.75, 90.00]	0.862
Large vessel occlusion (n/total, %)	192/360 (53.3)	222/276 (80.4)	< 0.001	239/359 (66.6)	148/242 (61.2)	0.203	396/617 (64.2)	23/25 (92.0)	0.008
**TOAST (n/total, %)**			0.002			0.312			0.592
Large-artery atherosclerosis	82/276 (29.7)	73/234 (31.2)		93/290 (32.1)	57/188 (30.3)		148/490 (30.2)	8/26 (30.8)	
Cardioembolism	89/276 (32.2)	104/234 (44.4)		114/290 (39.3)	66/188 (35.1)		187/490 (38.2)	11/26 (42.3)	
Small-vessel occlusion (lacune)	53/276 (19.2)	18/234 (7.7)		35/290 (12.1)	32/188 (17.0)		70/490 (14.3)	1/26 (3.8)	
Stroke of other determined etiology	6/276 (2.2)	4/234 (1.7)		8/290 (2.8)	2/188 (1.1)		9/490 (1.8)	1/26 (3.8)	
Stroke of unknown etiology	46/276 (16.7)	35/234 (15.0)		40/290 (13.8)	31/188 (16.5)		76/490 (15.5)	5/26 (19.2)	
Mortality (n/total, %)	0 (0.0)	63/308 (20.5)	< 0.001	16/387 (4.1)	35/260 (13.5)	< 0.001	53/658 (8.1)	10/31 (32.3)	< 0.001
**90-day mRS (n/total, %)**			< 0.001			< 0.001			< 0.001
0	159/382 (41.6)	0 (0.0)		124/387 (32.0)	32/260 (12.3)		158/658 (24.0)	1/31 (3.2)	
1	148/382 (38.7)	0 (0.0)		104/387 (26.9)	38/260 (14.6)		148/658 (22.5)	0 (0.0)	
2	75/382 (19.6)	0 (0.0)		44/387 (11.4)	29/260 (11.2)		73/658 (11.1)	2/31 (6.5)	
3	0 (0.0)	77/308 (25.0)		45/387 (11.6)	26/260 (10.0)		75/658 (11.4)	2/31 (6.5)	
4	0 (0.0)	136/308 (44.2)		45/387 (11.6)	82/260 (31.5)		124/658 (18.8)	11/31 (35.5)	
5	0 (0.0)	32/308 (10.4)		9/387 (2.3)	18/260 (6.9)		27/658 (4.1)	5/31 (16.1)	
6	0 (0.0)	63/308 (20.5)		16/387 (4.1)	35/260 (13.5)		53/658 (8.1)	10/31 (32.3)	
Early neurological improvement (n/total, %)	272/371 (73.3)	115/276 (41.7)	< 0.001	389/389 (100.0)	0 (0.0)	< 0.001	381/624 (61.1)	7/27 (25.9)	0.001
Symptomatic intracranial hemorrhage (n/total, %)	3/382 (0.8)	28/308 (9.1)	< 0.001	7/388 (1.8)	20/263 (7.6)	0.001	0 (0.0)	32/32 (100.0)	< 0.001

*n* number, *total* number of non-missing values of each variable, *p* p-value of Pearson’s Chi-squared test for categorical variables and Mann–Whitney U test for continuous variables, *IQR* interquartile range, *mRS* modified Rankin scale, *mmol/L* millimoles per liter, *min* minutes, *mmHg* millimeters of mercury, *LDL* low density lipoprotein, *HDL* high density lipoprotein, *NIHSS* National Institutes of Health Stroke Scale, *BP* blood pressure, *TOAST* trial of org 10172 in acute stroke treatment.

### Multivariate analysis of association between TyG index and mortality, neurological outcomes and intracranial hemorrhage

Results of the logistic regression analysis are shown in Table 3. Age, LVO status, hypertension and admitting NIHSS was significantly associated with mortality in univariate analysis and these variables were adjusted for in the multivariate analysis. On adjustment with these factors, the TyG index remained significantly associated with mortality (OR: 2.12, 95% CI: 1.39–3.23, p = 0.001).

**Table 3:** Association of TyG index with mortality, poor functional outcome, ENI and SICH.

		Multivariate analysis		
		OR	95% CI	*p*
Association of TyG index with	Mortality (*n* = 690)	2.12	1.39–3.23	0.001
	Poor functional outcome (*n* = 690)	1.41	1.05–1.90	0.022
	ENI (*n* = 649)	0.68	0.52–0.89	0.004
	SICH (*n* = 693)	0.90	0.50–1.64	0.738

Adjusted odds ratio (OR), 95% confidence interval (CI) and p-value calculated from logistic regression after adjusting for hypertension, age, admitting NIHSS and LVO status.

*OR* odds ratio, *95% CI* 95% confidence interval, *p* p-value, *TyG index* triglyceride-glucose index, *ENI* early neurological improvement, *SICH* symptomatic intracranial hemorrhage.

With respect to neurological outcomes, TyG index was significantly associated with poor functional outcomes (OR: 1.41, 95% CI: 1.05–1.90, p = 0.022) as well as poorer ENI (OR: 0.68, 95% CI: 0.52–0.89, p = 0.004). No significant association was observed between TyG index and SICH (OR: 0.90, 95% CI: 0.50–1.64, p = 0.738) (Table 3).

### Comparing ‘High TyG’ and ‘Low TyG’ patients using a TyG index cut-off point

**Figure 1:**
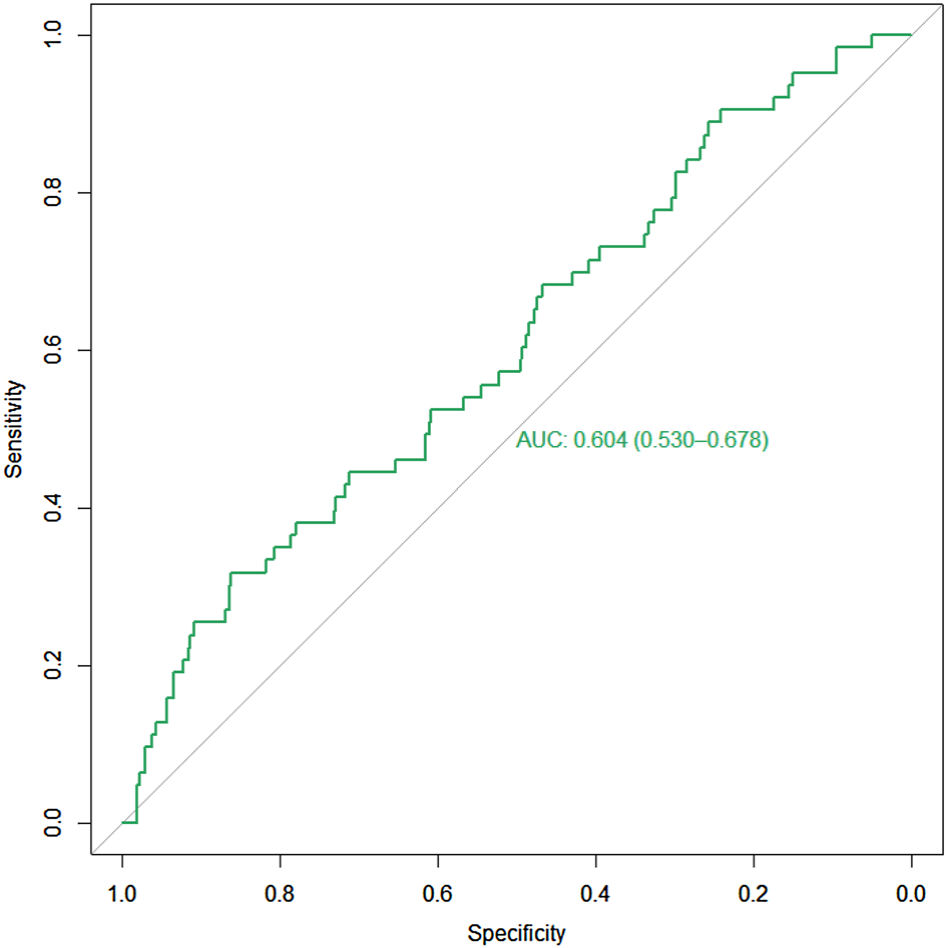
ROC curve for TyG index predicting mortality. TyG index of 9.15 was found to be a possible optimal cut-off point on the ROC curve (AUC: 0.604, 95% CI: 0.530–0.678, p = 0.007), predicting mortality with 33.3% sensitivity and 81.7% specificity. *ROC* receiver operating characteristic, *TyG index* triglyceride-glucose index, *AUC* area under the curve, *CI* confidence interval (De Long), *p* p-value calculated using Mann–Whitney U test.

Patients with a TyG index of < 9.15 were considered to have ‘Low TyG’ (n = 563) and patients with TyG index of ≥ 9.15 were considered to have ‘High TyG’ (n = 135), utilizing literature cut-offs for triglyceride (150 mg/dL or 1.7 mmol/L) and fasting glucose (7.0 mmol/L or 126 mg/dL)^30,31,34^. ROC curve analysis was employed to evaluate the association between the TyG index and the primary outcome of mortality, demonstrating an Area Under the Curve (AUC) of 0.604 (95% CI: 0.530–0.678, p = 0.007) (Fig. 1). The value of 9.15 derived as the cut-off for TyG index served as a possible optimal cut-off point on the ROC curve, predicting mortality with 33.3% sensitivity and 81.7% specificity. There were a higher proportion of deaths among ‘High TyG’ (n = 21, 15.6%) patients than ‘Low TyG’ patients (n = 42, 7.6%, p = 0.006). (Supplementary Table 1 [see Additional File 1]).

On multivariate logistic regression, after adjusting for clinical parameters (age, hypertension and LVO status) and stroke severity (admitting NIHSS), ‘High TyG’ index remained independently associated with mortality at 3 months (OR: 2.63, 95% CI: 1.39–4.99, p = 0.003), poor functional outcome (OR: 1.83, 95% CI: 1.17–2.85, p = 0.008) but not ENI (OR: 0.68, 95% CI: 0.45–1.02, p = 0.064), or SICH (OR: 0.95, 95% CI: 0.37–2.39, p = 0.909) (Table 4).

**Table 4:** Association of ‘High TyG’ (≥ 9.15) with outcomes including ordinal functional outcome mRS 0–6.

	‘High TyG’ (≥ 9.15) association with	Multivariate analysis		
		OR	95% CI	*p*
Logistic regression	Mortality (*n* = 690)	2.63	1.39–4.99	0.003
	Poor functional outcome (*n* = 690)	1.83	1.17–2.85	0.008
	ENI (*n* = 649)	0.68	0.45–1.02	0.064
	SICH (*n* = 693)	0.95	0.37–2.39	0.909
Ordinal logistic regression	Ordinal functional outcome (*n* = 690)	2.00	1.42–2.83	< 0.001

Adjusted odds ratio, 95% confidence interval and p-value calculated from logistic regression after adjusting for hypertension, age, admitting NIHSS and LVO status.

*‘High TyG’* triglyceride-glucose index ≥ 9.15, *OR* odds ratio, *95% CI* 95% confidence interval, *p* p-value, *poor functional outcome* dichotomous outcome of modified Rankin scale of 3 to 6, *ENI* early neurological improvement, *SICH* symptomatic intracranial hemorrhage, *ordinal function outcome* ordinal outcomes using modified Rankin scale 0 to 6.

**Figure 2:**
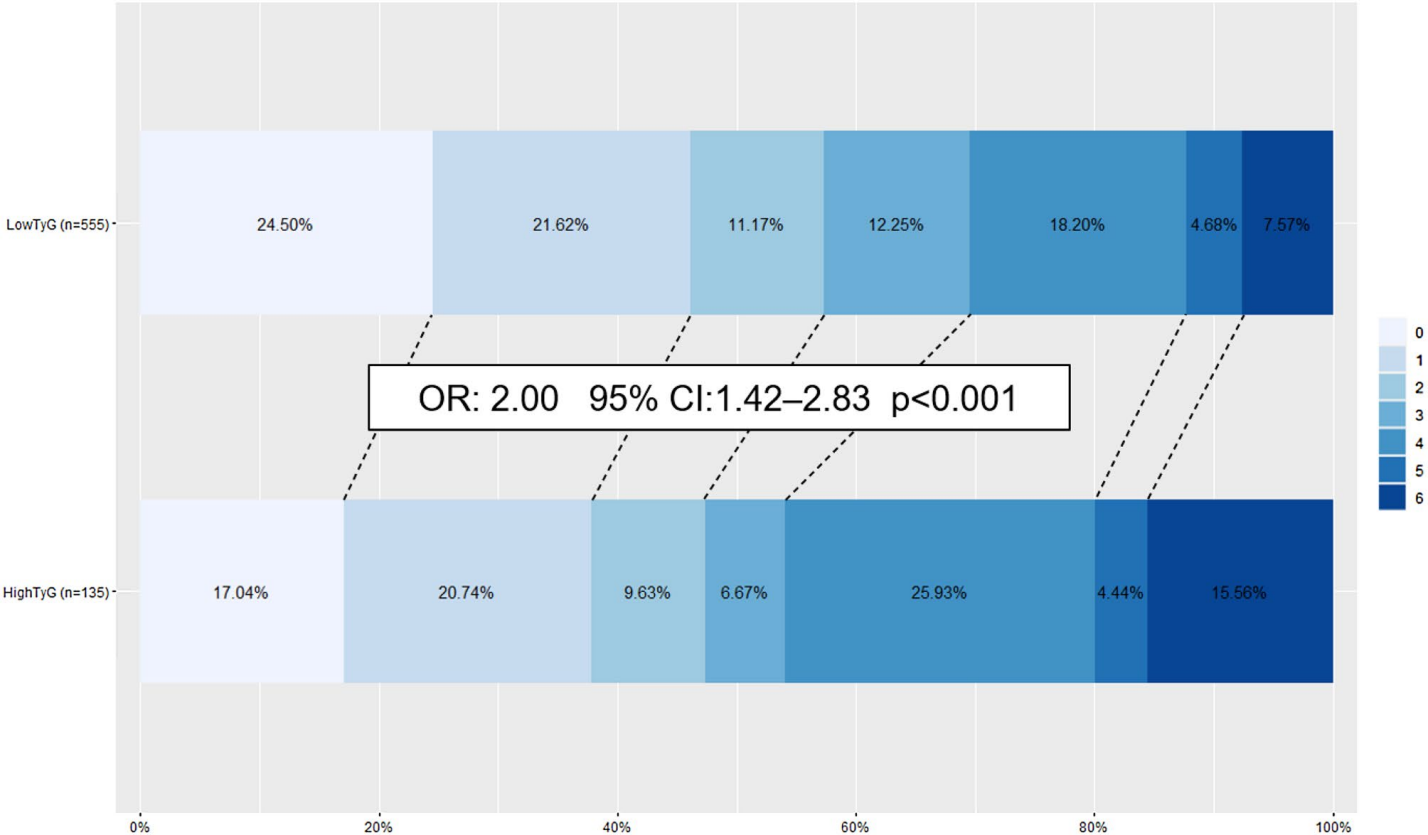
Ordinal shift analysis—comparison of mRS distribution between ‘low TyG’ and ‘high TyG’ patients. OR represents adjusted multivariate ordinal logistic regression of TyG index in model against ordinal mRS, adjusting for hypertension, age, admitting NIHSS and LVO status. Legend (right) demonstrates how mRS is represented by colours from 0 (in light blue) to 6 (in dark blue). Values with missing mRS values have been excluded from diagram. *OR* odds ratio, *95% CI* 95% confidence interval, *p* p-value calculated from ordinal logistic regression, *TyG index* triglyceride-glucose index, *mRS* modified Rankin scale, *NIHSS* National Institutes of Health Stroke Scale, *LVO* large vessel occlusion.

On ordinal shift analysis, patients with ‘High TyG’ were associated with having an unfavourable shift in ordinal mRS (OR: 2.00, 95% CI: 1.42–2.83, p < 0.001) after adjusting for clinical parameters (age, hypertension and LVO status), and stroke severity (admitting NIHSS) (Fig. 2).

### Subgroup analysis of TyG index and outcomes in patients with and without diabetes

Patients with DM had significantly higher median TyG index than patients without diabetes (8.96 [IQR: 8.55–9.41] vs 8.52 [IQR: 8.19–8.84], p < 0.001). (Supplementary Table 2 [see Additional File 1]) In a subgroup analysis of patients with diabetes (n = 214), the TyG index was not significantly associated with mortality (OR: 1.82, 95% CI: 0.95–3.49, p = 0.069), poor functional outcome (OR: 1.65, 95% CI: 0.99–2.75, p = 0.056), ENI (OR: 0.84, 95% CI: 0.54–1.30, p = 0.436) or SICH (OR: 0.46, 95% CI: 0.14–1.51, p = 0.201) in a multivariate model after adjustment for age, hypertension, admitting NIHSS and LVO status. In comparison, in patients without diabetes (n = 484), the TyG index was significantly associated with mortality (OR: 2.27, 95% CI: 1.15–4.47, p = 0.018), but not significantly associated with poor functional outcome (OR: 0.95, 95% CI: 0.63–1.43, p = 0.805), poor ENI (OR: 0.79, 95% CI: 0.54–1.17, p = 0.239) or SICH (OR: 1.20, 95% CI: 0.54–2.67, p = 0.649) in multivariate analyses (Table 5).

**Table 5:** Association of TyG index with outcomes in patients with and without diabetes.

		Multivariate analysis (patients with diabetes)				Multivariate analysis (patients without diabetes)		
		OR	95% CI	*p*		OR	95% CI	*p*
TyG index association with	Mortality (*n* = 211)	1.82	0.95–3.49	0.069	Mortality (n = 479)	2.27	1.15–4.47	0.018
	Poor functional outcome (*n* = 211)	1.65	0.99–2.75	0.056	Poor functional outcome (n = 479)	0.95	0.63–1.43	0.805
	ENI (*n* = 195)	0.84	0.54–1.30	0.436	ENI (n = 454)	0.79	0.54–1.17	0.239
	SICH (*n* = 211)	0.46	0.14–1.51	0.201	SICH (n = 482)	1.20	0.54–2.67	0.649

Adjusted odds ratio, 95% confidence interval and p-value calculated from logistic regression after adjusting for adjusting for hypertension, age, admitting NIHSS and LVO status.

*n* number, *OR* odds ratio, *95% CI* 95% confidence interval, *p* p-value, *TyG index* triglyceride-glucose index, *ENI* early neurological improvement, *SICH* symptomatic intracranial hemorrhage.

## Discussion

In this study, we observed an association between increased TyG index and mortality, poor functional outcome and poor ENI among post-thrombolysis AIS patients. Furthermore, on subgroup analyses, the TyG index was associated with an increased odds of mortality in patients without diabetes.

The TyG index was first introduced as a marker of insulin resistance in 2008 by Simental-Mendia to find a reliable alternative to HOMA-IR, with high sensitivity in detecting IR amidst non-diabetic subjects^32^. Compared to the HOMA-IR which utilizes glucose and insulin levels, the TyG index uses glucose and triglycerides values. Insulin is not routinely measured in clinical practice and the insulin assays are variable across platforms^35,36^. In comparison, serum triglyceride and plasma glucose levels are routinely performed in clinical practice and are well-calibrated across platforms^14^. The gold standard for evaluating IR is the hyperinsulinemic-euglycemic clamp (HEC), however, this labour-intensive and time-consuming procedure is should be performed in specialized metabolic research units requiring trained staff^37,38^. In 2010, it was then found that TyG index was comparable to and as reliable as the HEC test in assessing IR in a generic patient population including both diabetics and non-diabetics^14^. The TyG index has been shown to be a good predictor of metabolic health and early identification of IR, by identifying individuals with metabolic derangements^39^, and indexes involving the TyG index performed better than anthropometric measurements and traditional lipid markers to identify metabolic disease^12,40^.

The role of the TyG index in various cardiovascular and cerebrovascular diseases has also been increasingly studied. It is associated with arterial stiffness^41^, increased risk of cardiovascular disease and worse post-percutaneous coronary intervention outcomes^19^. In stroke, the TyG index has been associated with increased risk of stroke^22^, in-hospital mortality in critically-ill stroke^24^, and early recurrent ischemic lesions^25^. Baseline TyG index has been associated with increased mortality and neurologic deterioration in ischemic stroke^23^. However, studies have not been conducted to investigate the role of TyG index in patients post-thrombolysis, a key component of stroke management.

Calculation of the TyG index involves fasting glucose and triglycerides, both of which have been shown to have some association with stroke independently. Hyperglycemia in stroke is well known to be associated with poor survival and outcomes, increased infarct volume, increased risk of hemorrhagic conversion and reduced recanalization after IV-tPA^42,43^. While studies have not been conducted on the associations of post-thrombolysis outcomes with fasting triglycerides, the Reduction of Cardiovascular Events with Icosapent Ethyl–Intervention Trial (REDUCE-IT) study suggested that triglyceride lowering via the use of icosapent ethyl, a purified eicosapentaenoic acid (EPA) ethyl ester, significantly reduces the risk of composite cardiovascular outcomes and stroke^44^. However, there is evidence to suggest the TyG index is more strongly associated with cardiovascular and cerebrovascular outcomes than fasting glucose and triglycerides alone^18,45^. A large-scale prospective cohort study comprising 273,368 individuals found a significant association of the TyG index in cerebrovascular and ischemic stroke events, compared to the relationship of joint exposure of glucose and triglycerides scores with cerebrovascular disease which did not reach significance, recommending the TyG index to be a sensitive pre-diagnostic indicator for disease^18^. Furthermore, the TyG index is shown to be superior to fasting glucose or triglycerides in diagnosing metabolic syndrome and for diabetes prediction^17,46^. These argue for the TyG index’s utility as an effective way to assess outcomes in cerebrovascular disease as an appropriate measure of IR.

We observed worse mortality, neurological and functional outcomes in patients with high TyG index. A possible mechanism is that IR causes an increase in chronic proinflammatory cytokines and prothrombotic responses and endothelial dysfunction, exacerbating brain damage post stroke^47–49^. It has been suggested that the highly related concept of metabolic syndrome could increase resistance to thrombolysis due to an impaired fibrinolytic system or increased clot density^50^, and those with more metabolic syndrome components could increase the density of the clot structure and make it more resistant to lysis^51^. This suggests that identifying and targeting IR for patients to prevent future poor response to thrombolysis may be beneficial, but further studies will be required.

As there is a high prevalence of stroke patients who have concomitant diabetes in our local population (43.2%)^52^, and patients with diabetes are known to have poorer post-thrombolysis stroke outcomes compared to those without diabetes^53^, we proceeded to perform a subgroup analysis comparing the outcomes of patients with and without diabetes. Our study observed no association between TyG index and stroke outcomes in patients with diabetes, although the association with mortality and poorer functional outcomes was approaching statistical significance. There are several possible mechanisms for poor response to thrombolysis in diabetic patients—including vascular changes such as cerebral vascular endothelial dysfunction, arterial stiffness and thickening of capillary basal membrane^54^, hyperglycemia-induced overproduction of reactive oxygen species^55^, and increased and deregulated neuroinflammation, which could lead to worsened cerebral ischemia and incomplete recanalization after thrombolysis in a patient with diabetes^56,57^. These affect neurological outcomes through processes that may not be related to IR. Furthermore, in our study, subjects with diabetes were older, and had more cardiovascular risk factors including hypertension and hyperlipidemia. (Supplementary Table 2 [see Additional File 1]) Hence, after adjustment for these comorbidities in multivariate analysis, and accounting for other mechanisms that influence neurological outcomes in diabetes, the TyG index could exert less predictive effect on mortality and functional outcomes in a patient with established diabetes. Nonetheless, the associations of TyG index with mortality and functional outcomes were approaching statistical significance, which also may be related to the relatively small sample size of patients with diabetes in this study (n = 214).

Meanwhile, we found that higher TyG index was significantly associated with mortality but not functional outcomes, ENI or SICH after thrombolysis in patients without diabetes. IR has been associated with poor outcomes after ischemic stroke in patients without diabetes^58,59^, including in those who received thrombolysis^27^. The TyG index was also shown to be a good predictor of neurological worsening and stroke recurrence in AIS patients without diabetes^23^. Although our study did not replicate previous results in demonstrating an association of the TyG index with functional outcomes or ENI, this could be due to differing study populations and sample size. Nonetheless, our results imply that identifying and treating elevated IR even among AIS patients without diabetes could be important to improving post-thrombolysis stroke outcomes including mortality.

Emerging studies have suggested targeting IR as a therapeutic strategy to improve post-stroke outcomes using anti-diabetic drugs in both patients with and without diabetes. The Insulin Resistance Intervention after Stroke (IRIS) trial which involved 3876 patients, demonstrated that pioglitazone, an insulin-sensitizer, reduced the risk of recurrent stroke and myocardial infarction in patients without diabetes who had recent ischemic stroke or TIA^11^. Others observed associations between prior metformin use and better neurological outcomes in patients with AIS and those who received thrombolytic therapy^60^. A meta-analysis on glucagon-like peptide-1 (GLP-1) receptor agonists like liraglutide, which work by glucose-dependent insulin release, have shown benefits for primary stroke prevention^61^. Experimental studies also showed how GLP-1 agonists may exert neuroprotective effects including through mechanisms that decrease insulin resistance in the brain^62^. These results suggest that trials targeting IR using anti-hyperglycemic insulin-sensitising agents could be explored to further target IR in a post-thrombolysis population, possibly even in patients without diabetes with high levels of IR.

With respect to limitations, this was a retrospective cohort study where causality could not be established. We were not able to compare the TyG index with other markers of insulin resistance, such as the HOMA-IR or hyperinsulinemic-euglycemic clamp, as only data which had been collected on admission as part of routine clinical practice was used. Furthermore, we did not have sufficient data on height, weight, or waist circumference (WC) to compare other related TyG indices such as TyG-BMI (Body Mass Index) or TyG-WC Index. The retrospective nature of our cohort study performed in a single centre warrants future prospective studies to validate the role of the TyG index in predicting thrombolysis outcomes, and could include comparison with HOMA-IR or between patients with and without diabetes. Further studies could also investigate the effect of anti-diabetic agents which affect IR on post-thrombolysis stroke outcomes.

## Conclusion

In AIS patients who received tPA, the TyG index, a measure of insulin resistance, was significantly associated with increased 90-day mortality and poorer neurological and functional outcomes.

## Supplementary Information

Supplementary Tables.

## Data Availability

The data that support the findings of this study are available from National University Hospital but restrictions apply to the availability of these data, and so are not publicly available. Data are however available from the authors upon reasonable request and with permission of National University Hospital.
