# Supplementary material for: Association of triglyceride-glucose index with clinical outcomes in patients with acute ischemic stroke receiving intravenous thrombolysis
Source: Sci Rep. 2022 Jan 31;12:1596. doi: 10.1038/s41598-022-05467-6 (PMC8803886; doi:10.1038/s41598-022-05467-6)
Supplement: Supplementary file 1 — Supplementary Tables. [file 41598_2022_5467_MOESM1_ESM.pdf]

SUPPLEMENTARY INFORMATION

Supplementary Table 1. Baseline characteristics of patients with ‘Low TyG’ and ‘High TyG’

		Low TyG (<9.15)	High TyG (≥9.15)	*p*
		n =563	*n = 135*	
Age (median [IQR])		66.00 [55.00, 77.00]	63.50 [55.75, 72.25]	0.146
Male (n/total, %)		340/551 (61.7)	80/132 (60.6)	0.894
Ethnicity (n/total, %)				0.186
Chinese		339/497 (68.2)	85/120 (70.8)	
Malay		91/497 (18.3)	27/120 (22.5)	
Indian		31/497 (6.2)	3/120 (2.5)	
Others		36/497 (7.2)	5/120 (4.2)	
Co-morbidities (n/total, %)				
Smoking		84/255 (32.9)	24/57 (42.1)	0.246
Hypertension		333/419 (79.5)	95/111 (85.6)	0.188
Hyperlipidemia		252/368 (68.5)	74/93 (79.6)	0.049
Atrial Fibrillation		109/262 (41.6)	15/57 (26.3)	0.046
Diabetes Mellitus		130/563 (23.1)	84/135 (62.2)	<0.001
Laboratory findings				
Fasting glucose (mmol/L, median [IQR])		5.70 [5.10, 6.60]	8.00 [6.35, 11.80]	<0.001
HbA1c (%, median [IQR])		5.80 [5.50, 6.00]	7.00 [5.90, 9.05]	<0.001
Lipid Parameters				
Total cholesterol (mmol/L, median [IQR])		4.61 [3.87, 5.27]	5.12 [4.36, 5.94]	<0.001
LDL (mmol/L, median [IQR])		2.92 [2.22, 3.48]	3.08 [2.32, 3.76]	0.127
HDL (mmol/L, median [IQR])		1.16 [1.02, 1.39]	1.02 [0.91, 1.15]	<0.001
Triglycerides (mmol/L, median [IQR])		1.01 [0.78, 1.34]	2.19 [1.67, 2.74]	<0.001
Cholesterol HDL ratio (median [IQR])		3.85 [3.16, 4.66]	4.90 [4.24, 5.65]	<0.001
TyG Index (median [IQR])		8.50 [8.21, 8.76]	9.51 [9.30, 9.80]	<0.001
Stroke parameters				
Admitting NIHSS (median [IQR])		14.00 [8.00, 21.00]	14.00 [8.00, 20.00]	0.961
NIHSS at 24h (median [IQR])		6.00 [2.00, 14.00]	7.50 [3.00, 15.25]	0.122
Onset-to-Treatment time (min, median [IQR])		158.00 [118.00, 206.00]	148.00 [114.50, 201.50]	0.26
Admitting Systolic BP (mmHg, median [IQR])		151.00 [135.00, 166.00]	155.00 [140.00, 170.00]	0.072
Admitting Diastolic BP (mmHg, median [IQR])		82.00 [72.00, 91.00]	86.00 [74.75, 95.00]	0.063
Large Vessel Occlusion (n/total, %)		339/519 (65.3)	81/124 (65.3)	1
TOAST (n/total, %)				0.059
Large-artery atherosclerosis		117/410 (28.5)	40/107 (37.4)	
Cardioembolism		170/410 (41.5)	28/107 (26.2)	
Small-vessel occlusion (lacunae)		52/410 (12.7)	19/107 (17.8)	
Stroke of other determined etiology		8/410 (2.0)	2/107 (1.9)	
Stroke of unknown etiology		63/410 (15.4)	18/107 (16.8)	
Mortality (n/total, %)		42/555 (7.6)	21/135 (15.6)	0.006
90-day mRS (n/total, %)				0.01
	0	136/555 (24.5)	23/135 (17.0)	
	1	120/555 (21.6)	28/135 (20.7)	
	2	62/555 (11.2)	13/135 (9.6)	
	3	68/555 (12.3)	9/135 (6.7)	
	4	101/555 (18.2)	35/135 (25.9)	
	5	26/555 (4.7)	6/135 (4.4)	
	6	42/555 (7.6)	21/135 (15.6)	
Early Neurological Improvement (n/total, %)		327/533 (61.4)	62/119 (52.1)	0.079
Symptomatic Intracranial Hemorrhage (n/total, %)		26/562 (4.6)	6/135 (4.4)	1

Abbreviations- n: number, total: number of non-missing values of each variable, p: p-value of Pearson’s Chi-squared test for categorical variables and Mann-Whitney U test for continuous variables, IQR: Interquartile range, mRS: modified Rankin Scale, mmol/L: millimoles per liter, min: minutes, mmHg: millimeters of mercury, LDL: Low Density Lipoprotein, HDL: High Density Lipoprotein, NIHSS: National Institutes of Health Stroke Scale, OTT: Onset-to-Treatment Time, BP: Blood pressure, TOAST: Trial of Org 10172 in Acute Stroke Treatment

Supplementary Table 2. Baseline characteristics among patients with and without diabetes

	Patients without Diabetes *n=484*	Patients with Diabetes *n=214*	*p*
Age (median [IQR])	63.00 [54.00, 76.00]	68.00 [58.25, 77.00]	0.003
Male (n/total, %)	306/476 (64.3)	114/207 (55.1)	0.029
Ethnicity (n/total, %)			0.002
Chinese	309/432 (71.5)	115/185 (62.2)	
Malay	66/432 (15.3)	52/185 (28.1)	
Indian	24/432 (5.6)	10/185 (5.4)	
Others	33/432 (7.6)	8/185 (4.3)	
Co-morbidities (n/total, %)			
Smoking	88/258 (34.1)	20/54 (37.0)	0.799
Hypertension	272/366 (74.3)	156/164 (95.1)	<0.001
Hyperlipidemia	203/326 (62.3)	123/135 (91.1)	<0.001
Atrial Fibrillation	98/258 (38.0)	26/61 (42.6)	0.601
Diabetes Mellitus	0 (0.0)	214/214 (100.0)	<0.001
Laboratory findings			
Fasting glucose (mmol/L, median [IQR])	5.60 [5.00, 6.10]	8.00 [7.10, 9.95]	<0.001
HbA1c (%, median [IQR])	5.70 [5.50, 6.00]	6.60 [5.80, 8.00]	<0.001
Lipid Parameters			
Total cholesterol (mmol/L, median [IQR])	4.68 [3.95, 5.39]	4.69 [3.91, 5.30]	0.931
LDL (mmol/L, median [IQR])	2.93 [2.25, 3.50]	2.94 [2.25, 3.56]	0.819
HDL (mmol/L, median [IQR])	1.15 [0.98, 1.37]	1.10 [0.96, 1.29]	0.049
Triglycerides (mmol/L, median [IQR])	1.11 [0.83, 1.53]	1.19 [0.85, 1.70]	0.073
Cholesterol HDL ratio (median [IQR])	4.01 [3.24, 4.90]	4.24 [3.32, 5.00]	0.081
TyG Index (median [IQR])	8.52 [8.19, 8.84]	8.96 [8.55, 9.41]	<0.001
Stroke parameters			
Admitting NIHSS (median [IQR])	14.0 [8.0, 20.0]	16.0 [8.0, 21.0]	0.054
NIHSS 24h (median [IQR])	5.0 [2.0, 13.0]	9.5 [3.0, 18.0]	<0.001
Onset-to-Treatment time (min, median [IQR])	158.00 [120.00, 205.00]	149.00 [111.00, 204.00]	0.210
Admitting Systolic BP (mmHg, median [IQR])	150.00 [135.00, 166.00]	156.00 [138.00, 172.00]	0.011
Admitting Diastolic BP (mmHg, median [IQR])	82.00 [73.00, 91.00]	84.00 [74.00, 94.00]	0.142
Large Vessel Occlusion (n/total, %)	288/450 (64.0)	132/193 (68.4)	0.326
TOAST (n/total, %)			0.736
Large-artery atherosclerosis	114/372 (30.6)	43/145 (29.7)	
Cardioembolism	138/372 (37.1)	60/145 (41.4)	
Small-vessel occlusion (lacunae)	50/372 (13.4)	21/145 (14.5)	
Stroke of other determined etiology	7/372 (1.9)	3/145 (2.1)	
Stroke of unknown etiology	63/372 (16.9)	18/145 (12.4)	
Mortality (n/total, %)	31/479 (6.5)	32/211 (15.2)	<0.001
90-day mRS (n/total, %)			<0.001
0	125/479 (26.1)	34/211 (16.1)	
1	116/479 (24.2)	32/211 (15.2)	
2	50/479 (10.4)	25/211 (11.8)	
3	52/479 (10.9)	25/211 (11.8)	
4	87/479 (18.2)	49/211 (23.2)	
5	18/479 (3.8)	14/211 (6.6)	
6	31/479 (6.5)	32/211 (15.2)	
Early Neurological Improvement (n/total, %)	295/455 (64.8)	94/197 (47.7)	<0.001
Symptomatic Intracranial Hemorrhage (n/total, %)	21/483 (4.3)	11/214 (5.1)	0.791

Abbreviations- n: number, total: number of non-missing values of each variable, p: p-value of Pearson's Chi-squared test for categorical variables and Mann-Whitney U test for continuous variables, IQR: Interquartile range, mRS: modified Rankin Scale, mmol/L: millimoles per liter, min: minutes, mmHg: millimeters of mercury, LDL: Low Density Lipoprotein, HDL: High Density Lipoprotein, NIHSS: National Institutes of Health Stroke Scale, OTT: Onset-to-Treatment Time, BP: Blood pressure, TOAST: Trial of Org 10172 in Acute Stroke Treatment
